# Automatic Prediction and Annotation: There Are Strong Biases for Multigenic Families

**DOI:** 10.3389/fgene.2021.697477

**Published:** 2021-09-16

**Authors:** Catherine Mathé, Christophe Dunand

**Affiliations:** Laboratoire de Recherche en Sciences Végétales, Université de Toulouse, CNRS, UPS, Toulouse INP, Auzeville-Tolosane, France

**Keywords:** protein annotation, gene prediction, gene family, mis-prediction, mis-annotation

## Introduction

**Figure 1:**
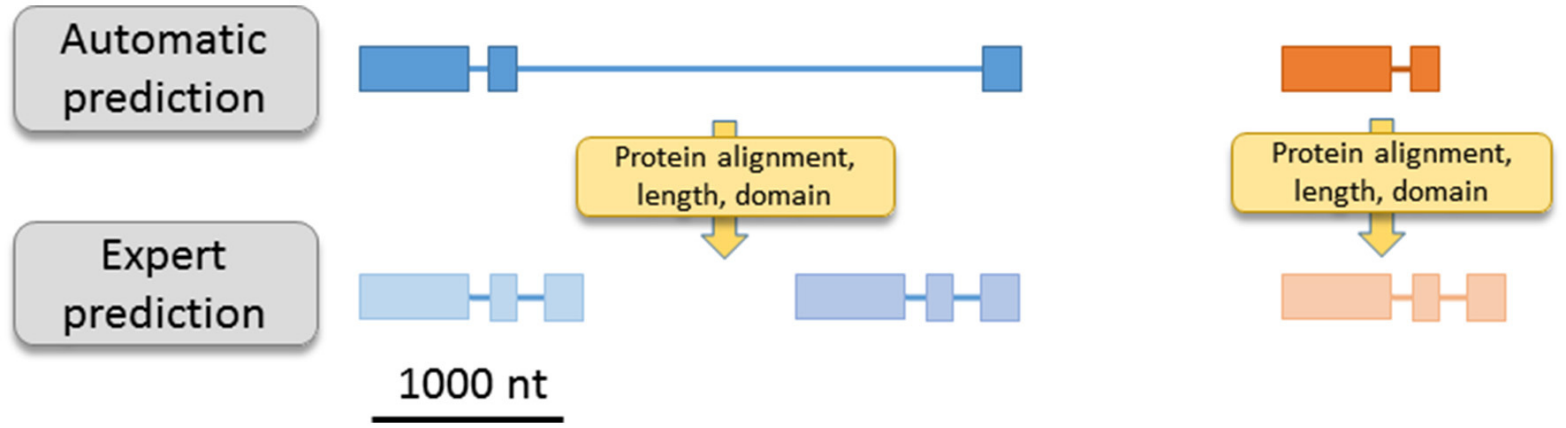
Illustration of two usual mis-prediction. For example, automatic prediction can generate one ORF result of two independent ORF fusion (left part) or partial ORF with a missing/partial exon (right part). Sequence homology, sequence length, domain detection… allow a large reduction of automatic bias.

In the last few decades, the explosion of genomic projects has produced huge sets of predicted genes and annotated sequences. The prediction of a gene structure can be defined as the capacity to determine the start and the stop of the gene as well as the positions of introns, if present. Despite the number of performant gene prediction programs combining *ab initio* and homology-based approaches (Mathe et al., 2002; Hoff and Stanke, 2015), the rate of mis-predicted genes is not negligible and can be due to several factors (Scalzitti et al., 2020). For example, unusually long introns, short exons or long genes can generate incomplete or partially predicted gene structure; short intergenic regions can lead to gene fusion; DNA sequencing errors (nucleotide deletions or insertions) introducing frameshifts can affect predictions; non-canonical splice sites, overlapping genes and genes located within introns are also a source of erroneous predictions. Due to high sequence identity and duplication rate, the risks of mis-prediction are exacerbated in the case of multigenic families (Figure 1, Fawal et al., 2014). In addition, protein annotation or function assignment, based on the presence of a hypothetical protein domain or on homology with known proteins, can also lead to an inappropriate annotation. The risk of mis-annotations is high for proteins containing multiple domains or small domain(s) common to several classes of proteins. For example, the PFAM domain PF07992 (Pyridine nucleotide-disulphide oxidoreductase) is detected in MonoDehydroAscorbate Reductases (MDARs), Glutathione Reductases (GRs), and in the Thioredoxin family (Trx) but does not discriminate between these three different families (Table 1). Mis-annotations are also observed for proteins belonging to superfamilies with conserved domain and large number of protein families and classes. As an example, 198 genes of the MYB superfamily have been detected in *Arabidopsis thaliana* (Yanhui et al., 2006), but the PFAM domain PF00249 (Myb_DNA-binding) does not discriminate between the R2R3-MYB, the R1R2R3-MYB, the MYB-related, and the atypical MYB families. In addition, the PF00249 entry also contains the SANT domain, which has a strong structural similarity to the Myb domain but is functionally divergent. Therefore, using this PFAM entry to extract MYB proteins returns many false positives (total of 326 sequences from *A. thaliana*).

**Table 1:** Illustration of PFAM domains diversities and specificities.

**PFAM**	**PFAM annotation**	**Targeted protein classes**	**Classes/subclasses abbreviation from Redoxibase**
PF00199	Catalase monofunctional (typical)	Catalase	Kat
PF06628	Catalase-related	Catalase	Kat
PF00255	Glutathione peroxidase	Glutathione Peroxidase	GPx
PF00141	Haem peroxidase	Class III peroxidases, Class II, and class I (APx, CcP, and CP)	Prx, CII, APx, CP, and CcP
PF00578	AhpC/TSA family	1-Cys or 2-Cys Peroxiredoxins Prx Q or BCP	1CysPrx, 2CysPrx, and PrxQ
PF08534	Redoxin	2-Cys Peroxiredoxins Prx II, Prx V	PrxIII and PrxV
PF03098	Animal haem peroxidase, An_peroxidase	Vertebrate peroxidase, Alpha-Dioxygenase, and Dual Oxidase	DiOx and DuOx
PF00210	Ferritin-like domain	Ferritin	Fer
PF13417	Glutathione S-transferase, N-terminal domain	Dehydroascorbate reductase	DHAR
PF07992	Pyridine nucleotide-disulphide oxidoreductase, Pyr_redox_2	MonoDehydroAscorbate Reductase, Glutathione Reductase, and Thioredoxin family	MDAR, GR, and Trx
PF02852	Pyridine nucleotide-disulphide oxidoreductase, dimerisation domain, Pyr_redox_dim	Glutathione Reductase	GR
PF01070	FMN-dependent dehydrogenase, FMN_dh	Glycolate Oxidase	GOx
PF01786	Alternative Oxidase	Alternative Oxidase	AOX, PTOX
PF02777	Iron/manganese superoxide dismutases, C-terminal domain, Sod_Fe_C	MnSOD and FeSOD	MSD and FSD
PF00080	Copper/zinc superoxide dismutase	Cu/ZnSOD and Cu chaperon for SOD	CSD and CCS
PF00462	Glutaredoxin (GLR)	Glutaredoxin (GLR)	4CxxC, GrxS, GrxC, CPF, ROXY
PF00085	Thioredoxin	Thioredoxin family, Thioredoxin M-type and Thioredoxin H -type	APR, CxxS, Lilium, Other Thioredoxin, TDX, TrxF, TrxH, TrxM, TrxO, TrxY
PF02298	Plastocyanin-like domain, Cu_bind_like	Blue-copper binding protein	ENODL, CRX, PNC, PC, STC, UCC
PF08022	FAD_binding_8	Dual Oxidase, Respiratory burst oxidase homolog and Ferric-chelate reductase	Duox, Rboh, and FRO
PF01794	Ferric reductase like transmembrane component, Ferric_reduct	Dual Oxidase, Respiratory burst oxidase homolog, and Ferric-chelate reductase	Duox, Rboh, and FRO
PF08030	Ferric reductase NAD binding domain, NAD_binding_6	Dual Oxidase, Respiratory burst oxidase homolog and Ferric-chelate reductase	Duox, Rboh and FRO

*The specificity of one PFAM domain can be low when it encompasses several protein families/classes/subclasses (gray cells). All PFAM descriptions are available from https://pfam.xfam.org/ (Mistry et al., 2021)*.

## ROS Gene Network, Contrasted Situations

Reactive Oxygen Species (ROS) are constitutively produced in plants during photosynthesis, respiration, and photorespiration but also produced in a control manner as signal or active molecules. In all cases, ROS homeostasis can be controlled by a large set of proteins described as ROS gene network (Inupakutika et al., 2016). Most of the proteins of this network are members of large superfamilies characterized by PFAM domains that are more or less specific. Indeed, one PFAM entry may encompass several classes or subclasses of proteins (Table 1, gray cells) and lead to mis-annotations.

**Figure 2:**
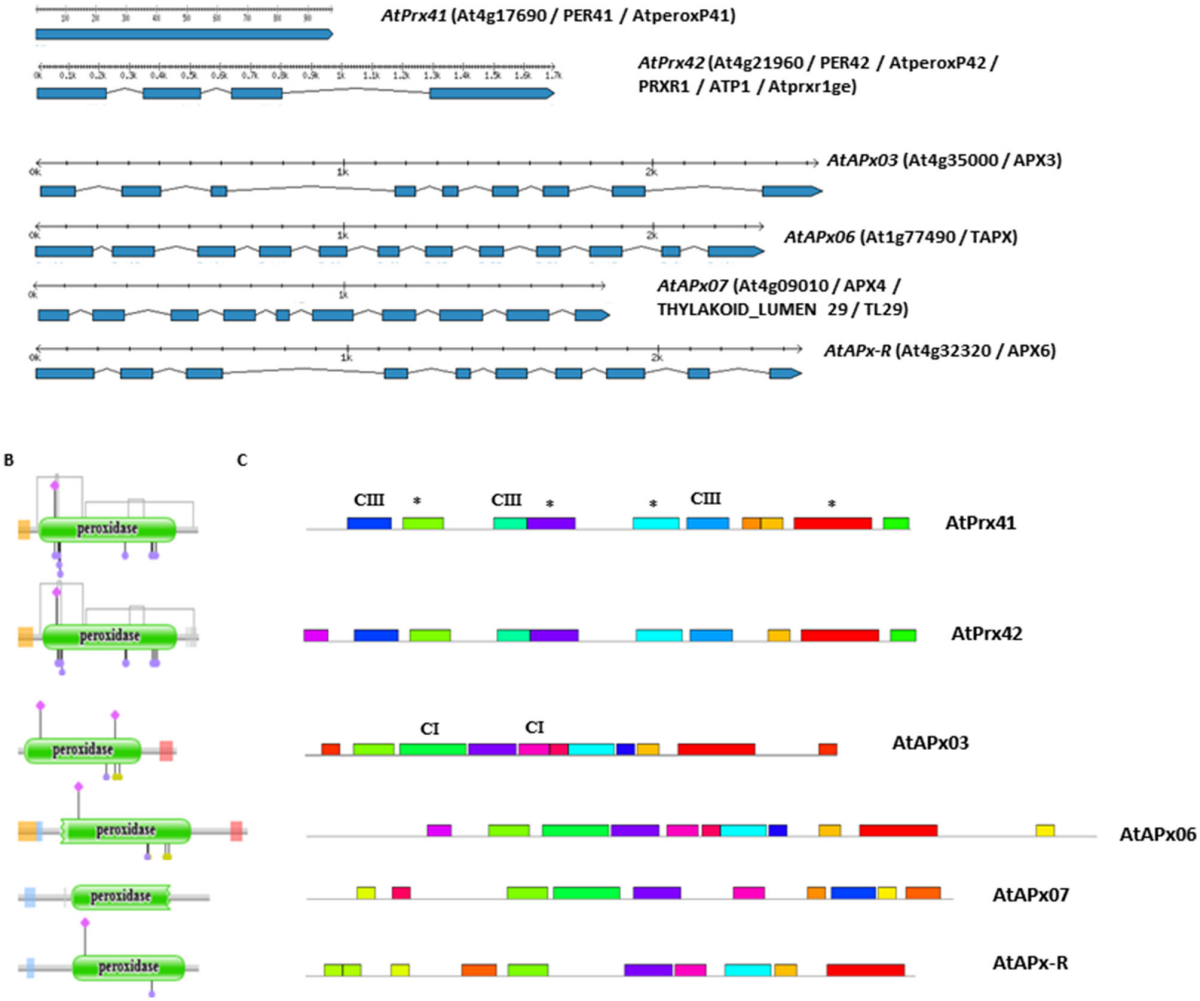
Two CIII Prxs (AtPrx41 and AtPrx42) and 4 APxs (AtAPx03, AtAPx06, AtAPx07, and AtAPx-R) sequences from *A. thaliana* have been compared using various available tools. **(A)** Structure intron/exon. Exons (blue boxes) and introns (gray lines) are drawn in scale. **(B)** Location of the unique PFAM domain PF00141 (green boxes), di-sulfide bridges (gray links) and conserved amino acid residues (pink diamonds, purple, and other disks) (Blázquez et al., 2003). **(C)** Conserved motifs in CIII Prxs and APxs. The MEME program was used to identify conserved motifs. The maximum number of motifs was set to 10, the optimum width of motifs to 15–50 amino acid residues (Brown et al., 2013). Each colored box represents a different motif found in CIII Prxs and/or APxs. CIII: motifs only found in CIII Prx protein sequences; CI: motifs only found in CI Prx protein sequences; ^*^: motifs in common between CIII and CI Prx protein sequences. All the sequences are available from the Redoxibase (Savelli et al., 2019).

Peroxidases, which belong to this network, participate in oxidation-reduction reactions using hydrogen peroxide (H_2_O_2_) as an electron acceptor and various substrates as electron donors. They may or may not contain a prosthetic group also called haem, justifying further subdivision into two major protein families, namely “haem peroxidases” and “non-haem peroxidases.” The haem peroxidases, such as the non-animal peroxidase family, are found in all kingdoms (Passardi et al., 2007). This family was first described thanks to structural homology (Welinder et al., 1992). It includes three classes of peroxidases: Class I (CI Prxs), Class II (CII Prxs), and Class III (CIII Prxs). This family is grouped under a unique PFAM entry (PF00141) (Table 1), which describes the conserved peroxidase domain (mainly the heam binding sites). This PFAM domain can extract most of the non-animal encoded sequences from any annotated genome, but unfortunately, it does not discriminate between the three classes (Figure 2B) and may produce erroneous annotations that require correction by experts. Over the past 5 years, 12 global phylogenetic and expression analysis of CIII Prxs from different plant species have been published, including four in 2020 (Ren et al., 2014; Wang et al., 2015; Cao et al., 2016; Moural et al., 2017; Duan et al., 2019; Wu et al., 2019; Yan et al., 2019; Zhu et al., 2019; Li et al., 2020; Xiao et al., 2020; Yang et al., 2020; Cai et al., 2021). These studies, based on available plant genomes, mostly contain incorrect predictions and annotations that may lead to erroneous or incomplete conclusions. Partial and longer sequences or pseudogenes were considered as complete sequences and APx sequences, which are CI Prxs, were annotated as CIII Prxs.

Plant NADPH oxidases, also known as Respiratory Burst Oxidase Homologs (RBOHs) catalyze the production of superoxide, ${\text{O}}_{2}^{-}$. They belong to a large gene family containing NADPH Oxidases (NOXs), found in animals and fungi, and the bifunctional proteins Dual Oxidases (DUOXs), present in animals. Due to the multi-domain organization, the family encompasses three PFAM accessions (PF08022, PF01794, PF08030) (Table 1). In addition, as the RBOH family is composed of a reduced number of copies (about 10), the risk of mis-annotation is reduced compared to CIII Prxs. Otherwise, the high number of introns, together with the short length of some introns and exons, are a source of mis-prediction. Since 2019, more than 10 articles dealing with the global phylogenetic and expression analysis of RBOHs from different plant species have been published (Cheng et al., 2013, 2019; Kaur et al., 2018; Chang et al., 2020; Wang et al., 2020; Yu et al., 2020). Despite their multi-domain composition and long length, few mis-predictions were detected. This may be due to the low duplication rate and to the low sequence conservation.

## Solutions to Improve Prediction and Annotation Errors

If this situation is extrapolated to all multigenic families (2,024 gene families in *A. thaliana* involving 17,481 genes) and to all available and annotated plant genomes (up to date, 134 publicly available from Phytozome, https://phytozome-next.jgi.doe.gov/), we are afraid that a hundred published studies already led to partial or incorrect conclusions.

The guarantee of an exhaustive and qualitative set of sequences is necessary to perform reliable studies, especially phylogeny, comparative genomic, and integrative analysis. Thus, efforts to provide high quality gene prediction and protein annotation are required, especially as mis-prediction and mis-annotation are rapidly amplified with subsequent articles that refer to incorrect results.

Is there a solution to reduce the rate of mis-prediction and mis-annotation in global analysis studies of large multigenic families? In the case of haem peroxidases, there are several cues to discriminate between CI APxs and CIII Prxs and to determine whether the gene predictions and protein annotations are accurate. (i) The number of gene copies is high and variable between species in CIII Prxs due to recent duplications, while it is low and conserved within the green lineage in APxs. (ii) The intron/exon structure (positions, number, and lengths of introns) is conserved in CIII Prxs (between none to three introns as illustrated Figure 2A with the two first lines) and distinct from that of APxs (between 8 to 10 introns as illustrated with the four last lines). Identification of conserved intron position and sequence alignment are powerful in discriminating between the two classes. (iii) The CIII Prxs contain conserved cysteines involved in 4 disulfide bonds whereas CI Prxs do not (Figure 2B). (iv) The protein size is characteristic as well as the highly conserved amino acids (pink diamonds, purple, and oher disks, Figure 2B) and the motifs of 15–50 amino acids defined with the MEME program (Bailey et al., 2015) (Figure 2C). (v) The CIII Prxs mostly contain a signal peptide, which targets them to the secretion pathway, whereas APxs are found in the various chloroplastic compartments or in the cytoplasm. Therefore, the combination of automatic prediction/annotation with a minimal expert control of sequence alignment should allow to verify the points (iii), (iv), and (v) and reduce the amount of erroneous predictions and annotations. Recently, new programs were developed to specifically address annotation of gene family taking into account intron conservation (Keilwagen et al., 2019) or preliminary search for a target domain (Kim et al., 2020). The generalization of these uses should be very helpful and significantly improve the sensibility and specificity of predictions.

## Conclusion

Expert annotations for large protein families and dedicated databases with manually verified proteins used as reference for prediction and annotation of additional genes are the solution. Currently, experts are already available for 166 families from The Arabidopsis Information Resource (TAIR) (https://www.arabidopsis.org/browse/genefamily/) and a few databases are dedicated to protein families. On the one hand, publications based on automatic annotations of genomes can still be done but, may lead to partial and error-prone conclusions. On the otherhand, expert annotation is a background work, time-consuming and not considered as an attractive task. This method has been experimented for some vertebrate genomes with the HAVANA group (https://www.sanger.ac.uk/group/vertebrate-annotation/) but it is hardly imaginable to extend it to the thousands of available genomes. However, expert annotation would reveal many incorrect predictions and annotations with a gain in terms of biological data, avoiding mis-interpretation in downstream analysis. An intermediary solution can be adopted, as in GENCODE (Frankish et al., 2021) which combines HAVANA manual expertise with automated annotation. In all cases, it remains the responsibility of the researchers to check the quality of annotation before drawing conclusions and formulating hypothesis. Despite the real progress made in annotating genomes as a whole, precautions are still crucial before interpretation, especially when gene families are involved.

## Author Contributions

CM and CD contributed to the writing. CD prepared the first draft and made the figure. All authors contributed to the article and approved the submitted version.

## Conflict of Interest

The authors declare that the research was conducted in the absence of any commercial or financial relationships that could be construed as a potential conflict of interest.

## Publisher's Note

All claims expressed in this article are solely those of the authors and do not necessarily represent those of their affiliated organizations, or those of the publisher, the editors and the reviewers. Any product that may be evaluated in this article, or claim that may be made by its manufacturer, is not guaranteed or endorsed by the publisher.
